# Regulation of *Drosophila* Hematopoiesis in Lymph Gland: From a Developmental Signaling Point of View

**DOI:** 10.3390/ijms21155246

**Published:** 2020-07-24

**Authors:** Wenwen Lan, Sumin Liu, Long Zhao, Ying Su

**Affiliations:** 1 Institute of Evolution & Marine Biodiversity, Ocean University of China, Qingdao 266003, China; lanvl311@gmail.com (W.L.); liusumin@stu.ouc.edu.cn (S.L.); 2 College of Marine Life Sciences, Ocean University of China, Qingdao 266003, China; 3 Fisheries College, Ocean University of China, Qingdao 266003, China

**Keywords:** *Drosophila melanogaster*, hematopoiesis, lymph gland, developmental signaling

## Abstract

The *Drosophila* hematopoietic system is becoming increasingly attractive for its simple blood cell lineage and its developmental and functional parallels with the vertebrate system. As the dedicated organ for *Drosophila* larval hematopoiesis, the lymph gland harbors both multipotent stem-like progenitor cells and differentiated blood cells. The balance between progenitor maintenance and differentiation in the lymph gland must be precisely and tightly controlled. Multiple developmental signaling pathways, such as Notch, Hedgehog, and Wnt/Wingless, have been demonstrated to regulate the hematopoietic processes in the lymph gland. Focusing on blood cell maintenance and differentiation, this article summarizes the functions of several classic developmental signaling pathways for lymph gland growth and patterning, highlighting the important roles of developmental signaling during lymph gland development as well as *Drosophila* larval hematopoiesis.

## 1. Introduction

Developmental signaling pathways control the vast majority of cellular processes during animal development. Several important signals, such as Wnt/Wingless (Wg), Hedgehog (Hh), and Notch, are repeatedly used to accurately, timely, and specifically instruct tissue/organ development [1,2,3,4]. These pathways share similarities in the principles of signal transduction. In different developmental contexts, individual signaling may exhibit complexity in the composition of pathway components, transducing events, or cellular functions. Therefore, the characteristics and functions of these developmental signaling pathways should be carefully illustrated in a given organ or tissue.

The *Drosophila* lymph gland, a larval specialized hematopoietic organ, has emerged as an excellent model system to study hematopoiesis. The lymph gland is retained throughout larval development and dissociates during metamorphosis [5]. As a site of definitive hematopoiesis, the larval lymph gland harbors both stem-like hemocytic progenitor cells (prohemocytes) and differentiated blood cells. Coordinated cell signals are required in the specification and regulation of lymph gland hemocytes. In addition, the lymph gland is known to be a vital organ in the defense system of *Drosophila* larvae upon an immune stimulus, such as wasp parasitism, starvation, and injury, which has been thoroughly reviewed [6,7,8]. In this review, focusing on the developmental process of lymph glands under normal conditions, we summarize the current understanding of the blood cell type, function, and patterning in the lymph gland, particularly highlighting the physiological roles of several classic developmental signalings in the control of cell proliferation and lineage determination during *Drosophila* larval hematopoiesis.

## 2. *Drosophila* Blood Cell Types and Functions

The hemocytes in *Drosophila* participate in the immune response through antimicrobial peptide production, phagocytosis, melanization, and encapsulation. At least three distinct types of terminally differentiated blood cells are produced from *Drosophila* hematopoiesis: plasmatocytes, crystal cells, and lamellocytes [9,10,11,12].

The most abundant hemocyte type is the plasmatocyte, which is relatively small, 8–10 μm in diameter, accounting for ~90–95% of hemocytes. They mainly have phagocytic and antimicrobial functions, similar to mammalian macrophages. Additionally, the plasmatocytes secret extracellular matrix (ECM) proteins to facilitate tissue formation and remodeling [13,14,15]. 

Crystal cells are slightly larger than plasmatocytes, ~10–12 μm in size, constituting 2–5% of total blood cells. The crystal cell is thus named because it possesses crystalline inclusions of prophenoloxidase (PPO) enzymes, which mediate melanin formation. As a non-phagocytic blood cell type, crystal cells function to release these melanization-related proteases to promote the innate immune response and wound healing [16,17]. Crystal cells can be clearly visible by the Black cell (*Bc*) mutation [18], which causes premature melanization of the crystalline inclusions.

The lamellocyte is large, flat, and irregular-shaped with a diameter of 15–40 μm. They are specialized in mediating the encapsulation and killing of pathogens too large to be phagocytosed. Accordingly, they are rarely observed in larvae under healthy conditions, but massively produced in response to an immune stimulus, such as wasp parasitization, injury, or mechanical stress. Different from plasmatocytes and crystal cells, lamellocytes are only found in larvae but not in embryos or adults, even post-wasp infestation [6,16,19]. 

## 3. Overview of *Drosophila* Hematopoiesis from Embryos to Adults

*Drosophila* hematopoiesis occurs in at least two waves in the development from embryos to adults [7]. The first wave of blood cell production occurs during embryogenesis. A cluster of cells derived from procephalic mesoderm (head mesoderm) gives rise to hemocyte progenitors, which differentiate to plasmatocytes and crystal cells. The plasmatocytes migrate throughout the embryos, while the crystal cells remain in the anterior midgut region [9]. These embryo-derived hemocytes persist into the larvae as either circulating cells in the hemolymph or sessile hemocytes that aggregate in the segmentally repeated epidermal-muscular pockets under the larval cuticle [19,20,21,22,23].

The second wave of hematopoiesis takes place in the larval stage. In addition to embryo-derived sessile hemocyte proliferation [22], a specialized hematopoietic organ, the lymph gland, develops along the anterior cardiac tube (dorsal vessel). The lymph gland grows in size, and the number of hemocyte within it increases by ~100-fold during larval development [24]. Under normal conditions, prohemocytes in the lymph gland give rise to plasmatocytes and crystal cells, both of which are released into the circulation as the lymph gland is broken during metamorphosis [5]. Upon immune stress, lamellocytes are produced in the lymph gland [25,26]. Besides this, the embryo-derived sessile hemocytes and circulating plasmatocytes also contribute to the lamellocyte production upon immune induction [20,21,27,28].

Both embryo and lymph gland-derived blood cells contribute to the adult hemocyte population [29,30]. It has been found that the majority of adult blood cells accumulate in the respiratory epithelia and fat body, and their numbers continuously decrease during aging [30,31]. In contrast to the knowledge gained from extensive studies in embryos and larvae, adult hematopoiesis remains largely elusive. In particular, the capacity of adult flies to produce new blood cells is currently under debate. It had been believed for a long time that hematopoietic activity is absent in adult flies [19,31]. Recently, Ghosh et al. identified active hematopoietic hubs at the dorsal side of the abdomen to support the existence of active hematopoiesis at adulthood [11]. They reported that the progenitors in these hubs undergo differentiation to mature blood cells. However, this conclusion was robustly argued by a more recent publication [30], in which no sign of blood cell proliferation or production was observed in adult flies even after bacterial infection. To resolve these contradictions, more thorough investigations in the future are expected.

## 4. *Drosophila* Lymph Gland

The *Drosophila* lymph gland is located in the dorsal aspect of the larva in association with the dorsal vessel, *Drosophila* heart [32], and is derived from embryonic thoracic mesoderm [24]. In contrast to embryonic blood cells, lymph gland precursor cells in embryos quickly proliferate only and do not differentiate. During dorsal closure, these precursor cells migrate dorsally and are positioned to flank the dorsal vessel in the thoracic segments. In the late embryo, these cells constitute the lymph gland with a single pair of lobes. The lymph glands from first-instar and early-second-instar larvae are largely populated by undifferentiated hemocytes. The differentiated blood cells first appear in the lymph gland during the mid-to-late second instar. In the third instar, the lymph gland contains undifferentiated prohemocytes, differentiating hemocytes, and mature blood cells. At the onset of pupariation, the lymph gland dissociates and bursts open to release blood cells into circulation [5]. 

### 4.1. Lymph Gland Zones

Although lymph gland development is initiated in the embryo, the establishment of zones and the majority of hemocyte maturation take place in the third larval instar. The lymph glands are structurally composed of several pairs of lobes, each separated by pericardial cells [33]. The anterior-most lobe, called the primary lobe, is the largest in size. The differentiation of blood cells occurs mainly in the primary lobes, whereas the other posterior lobes consist of undifferentiated hemocyte progenitors. 

**Figure 1:**
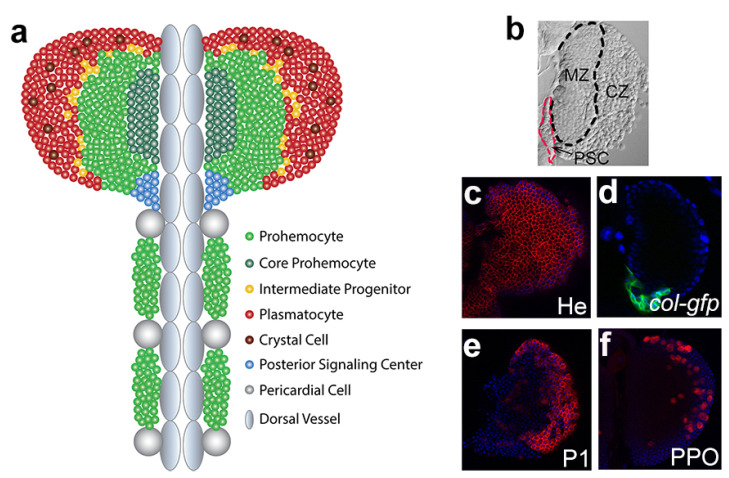
The lymph gland at the third instar larval stage. (**a**) A schematic diagram of the third-instar lymph gland. (**b**) A DIC image shows the morphological features of a primary lobe of the third-instar lymph gland. The black and red dashed lines outline the medullary zone (MZ) and posterior signaling center (PSC), respectively. The remaining area of the lobe is the cortical zone (CZ). (**c**–**f**) The immunofluorescence revealed by antibodies against Hemese (He), P1 antigen, or PPO, indicates pan hemocytes, plasmatocytes, and crystal cells, respectively, in red. The *col-gfp* transgene marks PSC in green. DAPI staining is blue.

When fully developed in third-instar larvae, the primary lobe can be divided into several zones based on cellular morphology, blood cell types, as well as distinct markers (Figure 1). A central zone, named the medullary zone (MZ), contains tightly packed blood cell progenitors (prohemocytes), which can be characterized by the expression of the JAK-STAT receptor *domeless* (*dome*). A periphery zone, termed the cortical zone (CZ), is rich in mature blood cells, which are loosely arranged and can be labeled by specific markers for plasmatocytes, crystal cells, and lamellocytes if there are any. Another crucial zone, called the posterior signaling center (PSC), is located at the posterior tip of the primary lobe and adjacent to MZ progenitors, specifically expressing the homeobox protein Antennapedia (Antp) [34]. Between MZ and CZ, there is an area termed the intermediate zone (IZ), where the cells undergo the transition from progenitors to specified blood cells, simultaneously expressing markers for both prohemocytes and early differentiated cells [24]. Recently, different subpopulations of Dome+ progenitors in the MZ were precisely defined with distinct markers. The most central progenitors that express *Tep4* or *collier* (*col*, also known as *knot*) are defined as the Core progenitors [35], and the neighboring progenitors that lack *Tep4* or *col* expression are named Distal progenitors [36].

During the lymph gland development, a small group of Dome-negative cells adjacent to the dorsal vessel were transiently observed in the first or second instar and identified as the founder cells of Dome+ prohemocytes [33,37]. This Dome-negative cell population in the first-instar lymph gland expresses several molecular markers that are associated with hematopoietic stem cells (HSCs) in vertebrates, and are multipotent to give rise to both progenitors and differentiated cells, and therefore has been termed HSCs [37]. Another clonal analysis also suggested the existence of HSCs in lymph glands [38]. Nevertheless, the important characteristics of stem cells, such as self-renewal or asymmetrical cell division, have not been clearly elucidated in these cells [37]. Thus, more evidence is expected to designate these cells as bona fide HSCs in *Drosophila* lymph glands.

### 4.2. Role of the PSC in the Lymph Gland

PSC cells exhibit high expression of Antp and the gene *col*, a *Drosophila* ortholog of mammalian Early B-cell Factor (EBF) [25,26,34], and produce multiple signaling molecules, such as Hh [34], Wg [39], Serrate (Ser, the ligand for Notch) [40], Decapentaplegic (Dpp) [41], and Pvf1 [a ligand for the platelet-derived growth factor (PDGF)] [42], all of which play critical roles in the maintenance of prohemocytes in the MZ during lymph gland hematopoiesis. Accordingly, the PSC has been established as an equivalent to the mammalian HSC niche. However, the requirement of PSC cells as well as the role of *col* in the PSC for progenitor maintenance have been challenged by recent studies [35,43], in which the genetic ablation of the PSC did not cause a loss of prohemocyte population. Later, additional studies repeatedly demonstrated that the signaling molecules sent out from the PSC, such as Hh and Ser, are indeed required to restrain hemocyte differentiation and maintain the MZ progenitors in an undifferentiated state [35,36,44], further supporting the critical role of the PSC as a hematopoietic niche. However, the role of *col* in the PSC has to be reconsidered. In contrast to PSC-expressing *col*, the *col* in the MZ has been revealed to act as an intrinsic regulator that controls the maintenance of prohemocytes, as knocking down *col* expression in the MZ led to a significant loss of prohemocytes and massive hemocyte differentiation [35,43], resembling the phenotype of a *col* mutant. In addition, it has been found that the MZ prohemocyte population is heterogeneous, containing both Col-positive and Col-negative cells [36,44]. The number of the Col-negative prohemocyte subpopulation is significantly reduced after PSC ablation, whereas the number of Col-positive prohemocytes is not affected by PSC manipulation [44]. The coexistence of PSC-dependent and PSC-independent subpopulations in the prohemocyte pool could be a clue to interpret the inconsistency reported for the role of PSC. 

Besides their role in normal developmental conditions, the essential role of PSC cells in controlling the proper differentiation of lamellocytes from progenitors after wasp parasitization has been repeatedly implicated [25,43,45,46]. Interestingly, the production of lamellocytes upon mechanical stress, such as squeezing fly larvae with forceps, is independent of the PSC cellular domain [47].

## 5. Regulatory Signaling during Lymph Gland Development

Developmental signaling is a major regulatory mechanism in the control of organ development. Many major developmental signaling pathways play important roles in regulating blood cell proliferation, differentiation, and patterning during the development of lymph glands. In the following section, we focus on several classic developmental signals and review their functions during the lymph gland hematopoietic process.

### 5.1. Notch Signaling Instructs Crystal Cell Fate Determination

Notch signaling is evolutionarily conserved from flies to mammals and plays a fundamental role in cell fate determination. The core component in this pathway is the single-pass transmembrane protein Notch. In the canonical pathway, the extracellular domain of Notch interacts with the ligand protein Delta or Ser located at the neighboring cell membrane, which triggers the cleavage of the Notch protein to release the Notch intracellular domain (NICD). Then, the NICD enters the nucleus and facilitates transcription factor Suppressor of Hairless [Su(H)] to activate target gene expression [48]. Notch activation through Ser is required for blood cell lineage specification and maintenance in the lymph gland.

The best characterized function of Notch signaling during larval hematopoiesis is to determine the crystal cell fate. Notch expression is ubiquitous throughout the third-instar lymph gland primary lobe [36,49]. Loss-of-function mutations in Notch result in a drastically decreased number of crystal cells [24,49,50], suggesting that Notch signaling is required for crystal cell differentiation. It has been demonstrated that Ser, but not Delta, is the Notch ligand for the specification of crystal cells [25,40,50,51]. Actually, the Notch pathway is employed several times during crystal cell formation. First, in Distal progenitors of the MZ, Notch pathway activity promotes the cell fate of undifferentiated cells towards crystal cells but not plasmatocytes. Overexpression of Notch, Ser, or Su(H) in the MZ dramatically increases the crystal cell number and blocks plasmatocyte differentiation [36]. Second, Notch signaling specifies the crystal cell precursors in the IZ and CZ regions by activating the expression of *lozenge* (*lz*), a transcription factor essential for commitment to crystal cell fate [40]. The expression of *lz* is diminished in the Notch or Su(H) loss-of-function mutant [40]. Third, Lz cooperates with Notch signaling to selectively activate a combination of crystal cell-related target genes, such as *klumpfuss* and *hindsight*, to further lock the cell fate towards the crystal cell and prevent the cells from adopting other cell fates [52]. Fourth, non-canonical Notch activation via stabilization of the Notch receptor in the endocytic pathway mediated by Hif-α (Sima in fly) facilitates the maturation and survival of crystal cells, which is independent of the Ser ligand [53].

Ser is highly expressed in PSC cells [25,34,40]. Notch signaling in the PSC mediated by Ser is critical in controlling the transcription of *col*, which is very important for maintaining the PSC cell number and signaling [25,41]. Interfering with Notch signaling, either through the expression of a dominant-negative form of Ser in the PSC or in Notch loss-of-function mutants, results in nearly complete loss of *col* transcription in PSC cells [26]. Knocking down Notch or Ser expression in the PSC is also able to non-cell-autonomously induce the formation of lamellocytes without wasp infestation [49], suggesting an inhibitory role of Notch signaling in lamellocyte differentiation under physiological conditions. The loss of *col* in the PSC blocks the formation of lamellocytes, even after parasitization [25]. Therefore, the lamellocyte differentiation induced by inhibiting Notch signaling is likely irrelevant with *col* in the PSC.

The Notch pathway is required cell-autonomously to maintain the Core progenitor in an undifferentiated state [36]. Silencing of Notch or Su(H) provokes a reduction in Core progenitors and an increase in mature hemocytes. Ser is required at the PSC to activate Notch for Core progenitor maintenance.

In addition, Notch signaling has been identified as the signature marker for HSC-like cells in first-instar lymph glands [37]. These cells express several indicators for active Notch signaling, including Notch, Su(H), and Enhancer of split mβ [E(spl)mβ]. The expression of Notch signaling activity is lost from these cells in the second instar. The function of Notch signaling in these cells remains elusive.

### 5.2. Hh Signaling Maintains the Prohemocyte Potency

Hh signaling is a conserved pathway that regulates cell proliferation, migration, and differentiation during development. The pathway is initiated by the binding of Hh with its receptor Patched (Ptc). G-protein-coupled receptor Smoothened (Smo) is activated to prevent phosphorylation and proteolysis of the transcription factor Cubitus interruptus (Ci), which eventually translocates into the nucleus to switch on target gene expressions [54]. In the lymph gland, Hh signaling is crucial for the maintenance of blood cell progenitors in MZ.

It is known that Hh is produced from signal-producing cells and functions as a morphogen to activate cellular signal transduction in the signal-receiving cells. Accordingly, *hh* is specifically expressed in PSC cells of the lymph gland, whereas the receptor Ptc and downstream Smo and Ci are highly expressed in the neighboring MZ region [34,44]. Depletion of *hh* in the PSC has no effect on the PSC itself but results in the loss of the MZ and an increase in the differentiated hemocyte number [34]. Similarly, the lymph gland with a loss-of-function Ci or Smo, or the expression of ectopic Ptc, shows a reduction in the prohemocyte number in the MZ [34,55,56]. Conversely, activating Hh signaling in the MZ by overexpressing a dominant-negative form of Ptc, or by inhibiting Suppressor of Fused (Sufu), a negative regulator of Ci transcriptional activity, causes a strong increase in blood progenitor cells in parallel to a reduction in mature hemocytes [56]. In contrast to manipulating the pathway downstream components, misexpression of *hh* in the MZ is not sufficient to expand the progenitor population, suggesting a possibility that Hh protein in non-PSC cells cannot function properly to activate the pathway due to an unknown disturbing mechanism. Collectively, these results indicate that Hh signaling originates from PSC cells and functions in the MZ to maintain prohemocytes in a hematopoietic precursor state and prevent their differentiation. Actually, not all the prohemocytes in the MZ are responsive to Hh signaling. A recent study further clarified that a particular subpopulation of MZ prohemocytes with Col-negative expression is sensitive to Hh signaling, and Hh signaling is required to maintain this prohemocyte subpopulation [44].

Hh production in PSC cells is precisely controlled by a combination of positive and negative regulatory mechanisms [57]. The GATA factor Serpent (Srp) is essential for *hh* expression in the PSC. The transcriptional regulators Su(H) and U-shaped (Ush) prevent *hh* expression in non-PSC cells of the lymph gland. Unlike the *sufu* mutant, misexpression of *hh* in the MZ is not sufficient to expand the progenitor population [56,57]. Interestingly, the expression of *ush* in prohemocytes requires Hh signaling. *hh* RNAi in the PSC, *Ci* RNAi in the MZ, *ptc* overexpression in the MZ, or loss of *smo* all result in a significant reduction in *ush* expression [44]. Thus, the Ush and Hh pathways collaborate through a regulatory loop to restrict *hh* expression within the PSC and maintain prohemocyte potency. In addition, Smo genetically interacts with Ush to block lamellocyte differentiation under normal conditions. Double heterozygotes for *ush*/*smo* exhibit a significant increase in the number of lamellocytes compared with that in single heterozygote for either *ush* or *smo* [44].

The Hh signaling pathway has been extensively studied in the wing system, where *col* is one of the well-established responsive genes for Hh signaling [58]. Conversely, *col* expression in the lymph gland may not be subject to Hh regulation. In lymph glands, *col* is highly expressed in PSC cells and at a low level in the Core progenitor cells of the MZ area. The *col* in the MZ and PSC functions independently to regulate progenitor maintenance and the hemocyte differentiation rate, respectively [35]. The *col* expression in the PSC is regulated by the Notch/Ser signal and can be inhibited by Jumu [26,59]. Although it is still unclear how *col* expression in the MZ is regulated, it has been revealed that *col* expression in the MZ and its function in those cells are independent of *hh* expressed by PSC cells [35].

### 5.3. Wnt/Wg Signaling Modulates Hemocyte Proliferation and Differentiation

Wnt/Wg signaling has been implicated in multiple developmental processes of metazoans, including cell fate specification, cell proliferation, and maintenance. The signal activated by Wg binding with its receptor is transduced through a series of cellular events and eventually protects Armadillo (Arm, *Drosophila* β-catenin) from degradation and promotes its translocation into the nucleus to activate target gene transcription [1]. In *Drosophila*, there are two receptor proteins for Wg ligand: Frizzled (Fz) and Frizzled 2 (Fz2). It has been shown that Wg signaling via either Fz or Fz2 is required to regulate MZ development, whereas Wg signaling mediated by Fz2, but not Fz, is responsible for controlling PSC cell and crystal cell proliferation [39,60].

The Wg protein is expressed throughout the entire lymph gland, and its expression level in MZ cells is much higher than that in CZ cells. Arm shows a similar expression pattern in the lymph gland [39]. It has been evidenced that Wg signaling in the MZ is able to maintain the MZ integrity and block prohemocyte differentiation. Impaired Wg signaling not only results in the mislocalization of Dome+ cells in the cortical area of the lymph gland, possibly through the downregulation of the cell adhesion molecule E-cadherin, but also initiates hemocyte differentiation, thus increasing the number of IZ cells. Conversely, elevated Wg signaling activities in the MZ, by overexpressing *arm* or *wg*, can inhibit the differentiation of prohemocytes, thus resulting in enlarged MZ and defective CZ regions.

It is known that the maintenance of the MZ prohemocyte pool requires signals from the PSC. The Wg pathway is able to positively regulate PSC cell proliferation through the proto-oncogene dMyc [39,41]. In addition, the maturation of crystal cells, but not plasmatocytes, is regulated by Wg signaling in the CZ. Overexpression of *wg* in crystal cell precursors (Lz+) results in an increased number of mature crystal cells, whereas downregulated Wg signaling in Lz+ cells causes a decreased number of crystal cells, suggesting that Wg signaling is able to promote the maturation and proliferation of crystal cells [39].

The ECM protein Tiggrin (Tig) has been identified as a downstream effector of Wg signaling [60,61]. In lymph glands, Wg signal inhibits the expression of *Tig*. Therefore, the Tig protein is highly expressed in plasmatocytes in the CZ, where the Wg signal is low, but not in MZ and crystal cells, where Wg activity is high. Consistent with its expression pattern, Tig likely functions only in the maturation of plasmatocytes, but not crystal cells and prohemocytes, as the *Tig* mutant shows a prematuration of plasmatocytes, while *Tig* overexpression blocks the maturation of plasmatocytes.

### 5.4. JAK-STAT Signaling Inhibits Prohemocyte Differentiation

The JAK-STAT signal transduction cascade is named from its two major components: Janus Tyrosine Kinase (JAK) and Signal Transducers and Activators of Transcription (STAT). The binding between the cytokine ligand and its single-pass transmembrane receptor induces conformational changes in the receptor and therefore triggers the phosphorylation of JAK and recruitment of STAT transcription factors. In *Drosophila*, several related proteins have been functionally characterized [62]: three cytokine ligands, Unpaired1–3 (Upd1–3); one receptor, Dome; one JAK kinase, Hopscotch (Hop); and one STAT protein, Stat92E. It has been well established that either local or systemic JAK-STAT signaling is closely associated with lamellocyte formation during an immune response [63,64]. Besides this, the lymph gland under healthy conditions utilizes both JAK-dependent and JAK-independent STAT signalings to maintain prohemocyte potency [42,65,66].

The receptor Dome is highly expressed in the MZ as a common marker of prohemocyte [26,33]. Consistently, as a signaling readout, the Dome-MESO-LacZ reporter is highly expressed in the MZ [26,65], indicating that JAK-STAT signaling is active in the MZ cells. The hypomorphic mutants for *dome* or *upd* show premature differentiation of plasmatocytes in the primary lobes of lymph glands [67], suggesting that JAK-STAT signaling in the MZ is important for keeping prohemocytes in an undifferentiated state. Consistently, JAK-STAT signaling is required for the expression of *ush*, which maintains the prohemocyte potency in the MZ [65].

JAK-STAT signaling is also involved in blood cell proliferation and differentiation in lymph glands. Larvae with a *hop* gain-of-function mutation always show hypertrophic lymph glands [68]. Consistently, ectopic expression of *hop* in the CZ, but not in the MZ, led to hyperplasia of the lymph gland, which requires Stat92E, Dome, and Upd3 [69]. In lymph glands with either the *hop* hypermorphic mutation or *hop* overexpression, an increased number of lamellocytes was also observed, even without parasitization [68,69]. Together, these results suggest that JAK-STAT signaling possesses the ability to enhance blood cell proliferation and induce lamellocyte formation in lymph glands. However, under physiological conditions, the JAK-STAT pathway might not be required for CZ development, as knocking down *hop* expression by RNAi in CZ cells resulted in no significant changes [69].

In differentiating hemocytes, STAT also mediates an “equilibrium signal” to maintain prohemocyte potency, most likely independent of the Dome-JAK pathway [42,66]. Stat92E is expressed throughout the entire primary lobe of the lymph gland [70]. Knocking down *stat92e* by RNAi specifically in the CZ results in the loss of the prohemocyte pool and elevates the number of plasmatocytes and crystal cells, whereas Stat92E loss-of-function outside of the CZ has no effect on blood cell differentiation [42,70]. The whole animal *stat92e* mutant reveals a loss of the MZ but an enlarged population of differentiated hemocytes [26], which is likely due to the loss of Stat92E function in the CZ. Further studies showed that STAT in the CZ functions downstream of PDGF/VEGF-like Receptor (PVR) signaling, which is activated by its ligand PVF1 from PSC cells, to promote the expression of Adenosine Deaminase-related growth factor (ADGF) [42,66]. A complex of ARF1 (Ras small GTPase) and Asrij (an endocytic protein) in endosomes provides a scaffold for STAT activation [67,71]. This PVF-PVR-STAT signaling cascade promotes lymph gland growth in the early stage and later cell-non-autonomously maintain the quiescence state of progenitors in the MZ [42,66].

### 5.5. BMP/Dpp Signaling Restricts PSC Size

Bone morphogenetic proteins (BMP) belong to the Transforming growth factor beta (TGFβ) superfamily. The BMP signal pathway is initiated by the BMP ligand binding with a type II receptor, which recruits and phosphorylates a type I receptor. Then, the type I receptor phosphorylates the SMAD protein and eventually activates target gene expressions in the nucleus. In *Drosophila*, there are two BMP ligands, Decapentaplegic (Dpp) and Glass bottom boat (Gbb); two type I receptors, Thickveins (Tkv) and Saxophone (Sax); two type II receptors, Wishful thinking (Wit) and Punt; one SMAD protein, Mother against Dpp (Mad); and multiple target genes, such as *daughters against Dpp* (*dad*) [72]. It has been demonstrated that BMP signal transduction is mainly mediated by the binding of the ligand Dpp to Tkv/Wit receptors in *Drosophila* lymph glands [41].

Dpp signaling is specifically activated in the PSC and is required for controlling its size during *Drosophila* larval hematopoiesis [41]. The activity of the BMP/Dpp signaling pathway can be reflected by the phosphorylation of the Mad protein and the expression of target genes. In wild type lymph glands, a high level of the phosphorylated Mad protein and target gene *dad* expression are both detected specifically in PSC cells, indicating that Dpp signaling is active in the niche. Blocking Dpp signaling either by using *dpp*, *tkv*, or *wit* mutants or by overexpressing a dominant-negative form of *tkv* significantly increases the number of PSC cells, suggesting that Dpp signaling negatively regulates PSC cell numbers. As PSC maintains prohemocytes, the loss of Dpp signaling in PSC cells results in a correlatively enlarged prohemocyte pool and impaired hemocyte differentiation. Furthermore, Dpp signaling maintains the low number of PSC cells by repressing dMyc expression in the PSC. The epistatic analysis indicated that Dpp signaling antagonizes Wg activity in the control of PSC cell numbers. Dpp signaling in the PSC is dependent on the local expression of *col*, as Dpp signal activity is lost when *col* expression is repressed in the PSC cells. In addition, Dpp signaling in the PSC can be enhanced by the Robo signal, which is activated by the glycoprotein Slit secreted from the dorsal vessel [73].

In addition to the cell-autonomous function of controlling PSC size, the Dpp signal is active in the PSC during the first instar larval stage and serves as a niche signal to maintain HSC-like cells, which are the founder cells of Dome+ prohemocytes in the MZ [37]. The phosphorylated Mad protein is enriched in these HSC-like cells. The knockdown of *dpp* in the PSC during the first instar stage or depletion of Mad in these HSC-like cells results in the loss of these HSC-like cells, and eventually drastically reduces the number of prohemocytes and the overall size of the lymph gland in the third instar larval stage. Notably, the PSC cell number did not change upon these manipulations, suggesting that the Dpp signal activity in the early larval PSC is not required for the development of the PSC itself.

Besides the signals mentioned above, many other pathways also play important roles during normal lymph gland development. The Fibroblast Growth Factor (FGF) ligands, Pyramus and Thisbe, and FGF receptor, Heartless, are expressed at high level in Dome+ progenitors, and have been found to facilitate blood cell differentiation in lymph glands [74]. Inhibition of FGF signaling in these prohemocytes by overexpressing a dominant-negative form of *heartless*, *thisbe* RNAi, or *pyramus* RNAi enlarges the MZ prohemocyte pool and decreases the differentiated hemocyte population. On the other hand, the enforced expression of FGF ligands or receptor results in a loss of Dome+ progenitor cells and an increased proportion of differentiated hemocytes in the primary lobes of lymph glands. This FGF-induced differentiation of prohemocytes also takes place in the second lobes, which normally harbor undifferentiated hemocytes. All these results indicate that FGF signaling is required for hemocyte differentiation and also sufficient to induce progenitor cell differentiation. The Hippo pathway, an evolutionarily conserved regulator of organ growth and cell fate determination, is known to regulate hematopoiesis by restricting hemocyte proliferation and differentiation in *Drosophila* lymph glands. Lymph glands lacking the downstream Hippo pathway kinase Warts are larger in size and filled with differentiated cells as a result of hemocyte overproliferation and premature differentiation [75,76]. The key effector of the Hippo pathway is the protein Yorkie, which is directly phosphorylated by Warts. Yorkie functions as a transcription co-activator, but its phosphorylation by Warts blocks its function by locking it in the cytoplasm. Yorkie and its binding partner, the transcription factor Scalloped, function together to regulate crystal cell formation in a cell-autonomous manner by transcriptionally activating *lz* expression in crystal cell progenitors [75] or in a non-cell-autonomous manner by promoting Ser expression in a subset of cortical area cells to instruct the neighboring Notch-positive cells to commit to the crystal cell fate [51,77]. Insulin signaling and downstream Target of Rapamycin (TOR) signaling are also known to stimulate cell proliferation via dMyc in the PSC [78,79,80,81,82], and are essential for progenitor maintenance [78,83,84].

## 6. Concluding Remarks

**Figure 2:**
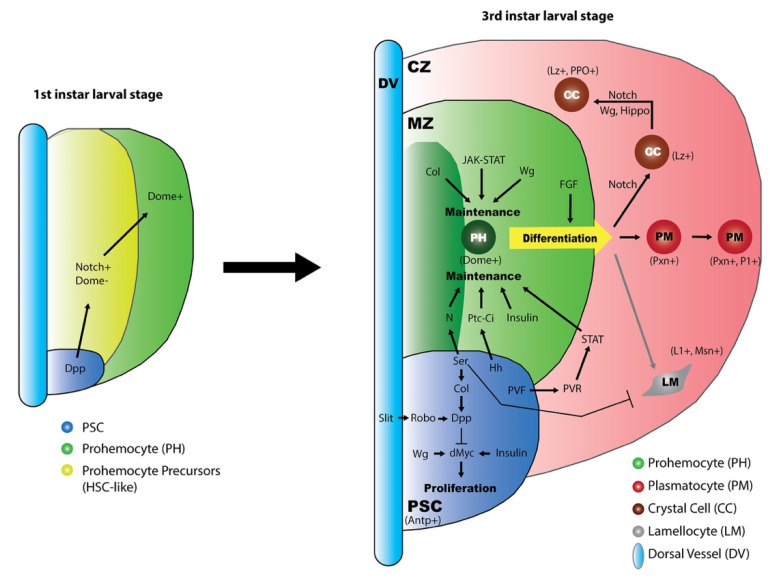
The regulation of lymph gland development by developmental signalings.

*Drosophila melanogaster* is a classic model organism that has made important contributions to genetic research. Recently, the *Drosophila* lymph gland has emerged as an excellent model system to study hematopoiesis. Undifferentiated progenitors and multiple types of differentiated blood cells coexist in the lymph gland, which allows researchers to address many questions regarding cell lineage and the signals that mediate blood cell progenitor maintenance, proliferation, and differentiation. Many classic development signaling pathways have been demonstrated to play vital roles during lymph gland hematopoiesis (Figure 2). In brief, the PSC in the lymph gland provides signal molecules, such as Hh, to activate signaling pathways in the neighboring MZ. The progenitors in the MZ exhibit high activities of local signals, such as Wg, JAK/STAT, and Col. These PSC-derived and MZ local signals, together with the equilibrium signals from differentiating cells in the CZ, such as PVR and downstream STAT, collaborate to maintain the progenitor cells in a quiescent state and/or regulate their differentiation. The differentiating blood cells require Notch signaling, which cooperates with Wg signaling and Lz, to develop towards crystal cell fate. To date, huge progress has been achieved in understanding the functions of developmental signaling pathways during larval hematopoiesis; however, their cross-regulation remains largely undefined.

Signaling events occur at precise locations and times to properly control animal development. Determining the spatiotemporal activity and function of developmental signals is critical for understanding the specific developmental process. During larval lymph gland hematopoiesis, many developmental signaling pathways play roles in specific areas and/or at particular times. For example, in the PSC, Dpp functions as a niche signal to maintain HSC-like cells during the early larval stage and controls the PSC cell number in the late larval stage. Instead, the role as a niche signal for MZ progenitor cell maintenance is taken by Hh signaling in the third-instar lymph gland. In the past decades, studies in the *Drosophila* lymph gland have benefited a lot from powerful experimental tools, such as versatile genetic approaches and lineage-tracing methods. Recently, many new techniques in *Drosophila* have been reported for monitoring and perturbing signaling molecules, such as the kinase translocation reporter (KTR)-based sensors, which enable the live-cell measurement of kinase activity at a single-cell level [85], and optogenetics and imaging techniques, which have been successfully utilized to study the function of cell signaling [86,87,88] or modulate cell contractility during embryogenesis [89]. These new techniques are promising for their application in future studies to address specific questions relevant to the spatiotemporal control of lymph gland hematopoiesis.

It is known that *Drosophila* hematopoiesis employs many conserved molecular strategies. For example, the *Drosophila* plasmatocytes are considered homologous to macrophages in humans, and the crystal cells and lamellocytes in *Drosophila* have functional similarities to platelets and giant cells in humans. Strikingly, many signaling pathways and transcription factors that control *Drosophila* hematopoiesis play comparable functions in mammals. Therefore, fully understanding the principles that control *Drosophila* hematopoiesis will definitely shed light on human hematopoiesis in further studies.
